# “Reimaging a triage system with midwives, for midwives”: exploring preferences for a midwife-Led triage system in South Africa through a user-centered approach

**DOI:** 10.3389/fdgth.2026.1720043

**Published:** 2026-03-20

**Authors:** Mxolisi Welcome Ngwenya, Livhuwani Muthelo, Melitah Molatelo Rasweswe, Tshepo Ntho, Tebogo Maria Mothiba

**Affiliations:** 1 Department of Nursing Science, University of Limpopo, Polokwane, South Africa; 2 Office of the Deputy Vice-Chancellor of Research Innovations and Partnerships, University of Limpopo, Polokwane, South Africa

**Keywords:** codesign, midwife-led, midwives, triage system, user-centered design

## Abstract

**Introduction:**

Triage in the maternity unit is critical to ensuring the delivery of timely and appropriate care. It is regarded as an initiative to reduce maternal mortality by accelerating the provision of appropriate care at the appropriate time. However, maternity units in South Africa lack standardized triage systems. Most pregnant women often wait for hours and days upon arriving at the hospital. This delay could prove costly for complicated pregnancies and childbirth. Therefore, this paper aims to explore the preferences and needs of midwives for a midwife-led triage system in South Africa.

**Methods:**

A user-centered design approach underpinned this study, focusing on the requirement gathering stage to fully understand the needs and preferences of midwives for an ideal midwife-led triage system. A sequential exploratory research design within the user-centered design framework was used. Experienced midwives were selected to participate in the study. Semistructured interviews and surveys were used for data collection, which were then analyzed using thematic analysis and descriptive statistics.

**Results:**

The study found that midwives had different needs and preferences for an ideal digital system. Two principal themes emerged from data integration, namely, triage contents and features of the midwife-led triage system and the application and functionality features of the midwife-led triage system. The study underscored the importance of including clinical profiles, patient profiles, clinical histories, and the designated area for subjective data such as chief complaints. In addition, the midwives expressed the need for the digital triage system to share information and be linked to primary healthcare facilities and laboratories to access patient results. They also highlighted the need for the system to provide clinical management guidance by incorporating maternal guidelines, addressing concerns about poor skills and incompetence among some midwives. The study underscored that preferences of midwives are shaped by their experience and the existing environment.

**Conclusion:**

Therefore, this paper suggests that the preferences, needs, and ideas of midwives should be integrated into the digital triage application to create a new quality triage system for maternity units in South Africa. Furthermore, relevant stakeholders should provide the resources required to navigate a smooth triaging process of patients with the midwife-led system.

## 1 Introduction

Access to timely, safe, and appropriate maternal healthcare remains a fundamental human right and a critical determinant of maternal and neonatal outcomes. Despite notable global progress in reducing maternal and perinatal mortality, South Africa continues to face persistent challenges in providing high-quality maternal health services (1, 2). For this reason, key elements of effective maternal healthcare have been implemented, including structured triage systems, which are essential for the systematic assessment, prioritization, and referral of expectant mothers according to the severity of their clinical condition (3). Triage systems help streamline service delivery, prevent care delays, and optimize maternal and neonatal outcomes, especially in high-volume or resource-constrained settings.

**Figure 1:**
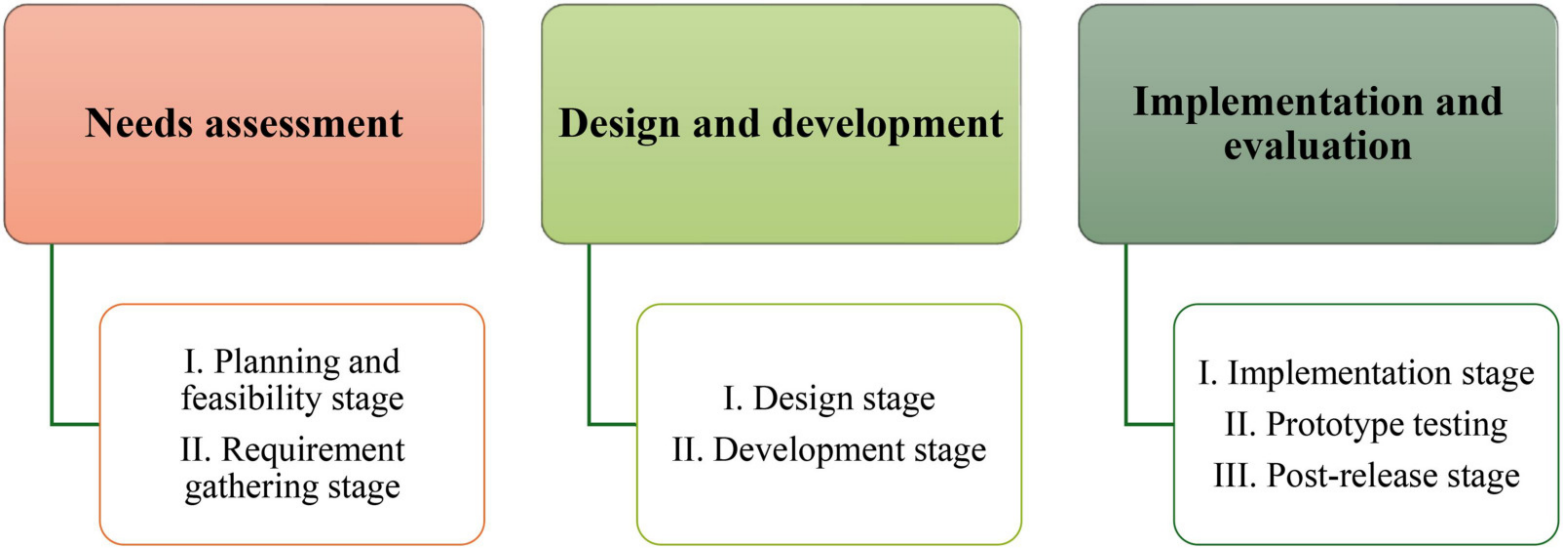
User-centered design process (15).

The World Health Organization underscores the importance of early risk identification and timely referral as part of the continuum of maternal and newborn care (4). Furthermore, Sustainable Development Goal (SDG) 3 explicitly calls for the reduction of global maternal mortality to less than 70 per 100,000 live births by 2030, emphasizing the need to strengthen health systems and ensure universal access to quality care. In alignment with these global priorities, South Africa's Maternity Care Guidelines advocate efficient, respectful, and evidence-based management of expectant mothers at all levels of care (5).

However, a critical gap in South African maternal care includes the lack of standardized triage systems (6). Current practices are frequently fragmented, inconsistently implemented, and heavily dependent on individual clinical judgment. Notably, these factors increase the likelihood of delays in the emergency response contributing to adverse maternal and neonatal outcomes (7). An examination of the literature highlights that midwives often lack the tools, designated space, protocols, or support systems to perform triage consistently and effectively (3, 8, 9). Therefore, this contemporary study ventures into codesigning a triage system to assist midwives when triaging expectant mothers. By engaging directly with midwives using a user-centered approach, this study explores preferences, insights, and lived experiences of midwives in triaging expectant mothers within maternity settings. This participatory design approach recognizes midwives as critical stakeholders whose knowledge and expertise can inform the development of practical, context-specific solutions.

This study builds upon the foundational premise that sustainable improvements in maternal healthcare require collaborative innovation, including cost-effective approaches (10). Rather than adopting externally developed frameworks that may not align with local realities, this study aims to codesign a triage system with midwives for midwives. By codesigning with midwives, the study ensures that the resulting midwife-led triage system is contextually relevant, practically usable, and widely acceptable. Equally important, a midwife-led triage system is crucial, especially where systemic limitations often hinder the delivery of timely and effective maternal care (6, 11). Therefore, the proposed midwife-led triage system has the potential to improve clinical decision-making, streamline emergency response, and ultimately support midwives to provide high-quality and equitable care. Importantly, the findings of this study support the National Digital Health Strategy for South Africa (2019–2024) by promoting timely access to maternal care, empowering midwives with effective triage tools, and improving the delivery of services (12).

## 2 Methods

### 2.1 Study design and setting

The study was carried out in district hospitals in Mpumalanga province, South Africa. This study was underpinned by a user-centered design (UCD) approach using a sequential exploratory research method. Adopting a mixed methods approach within the UCD approach allowed the authors to gather a deeper and better understanding of the phenomenon of interest by providing a clear picture of the preferences and needs of midwives for the triage system for women who present in selected public maternity units. This helped limit the weaknesses of qualitative and quantitative design as single methods and expose their strength as a mixed methods design in addressing complex research questions (13, 14).

### 2.2 Design process

A UCD approach was adopted to codesign a midwife-led triage system to scale up the triage practices of midwives (15). This approach enabled the authors to deeply interact with the target users to create digital and technological solutions that are grounded in information about people who will be using them and the context in which they will be deployed (16, 17). UCD emanates from human–computer interaction, which focuses on how humans relate to computing products. The UCD approach directly involves the safety, efficiency, effectiveness, and user satisfaction of the digital initiative (18, 19). The iterative process of the UCD approach along with its stages directed the exploration and needs assessment, data organization, and data collection throughout the period of the study. The iterative process of UCD is illustrated in Figure 1. However, this paper presents only the requirement stage of the needs assessment step.

#### 2.2.1 Needs assessment

##### 2.2.1.1 Planning and feasibility stage

This stage is regarded as the plan of action stage and mainly focuses on identifying the user of the intervention, their context of use, as well as their needs and preferences (15). The primary users identified in this study were midwives, with a specific focus on addressing the need to improve their triage practices. Using mixed methods, a baseline investigation of the triage practices and challenges of midwives was conducted. This was to determine the need for and necessity of the midwife-led triage system. The empirical findings obtained in the planning and feasibility analysis were essential to build a foundation for the requirement gathering stage (6, 20, 21).

#### 2.2.2 Requirement gathering stage

This paper focuses on the requirement gathering stage. It focuses on the identification of the needs and preferences of midwives to inform the design and development of a midwife-led triage system to assist midwives in triaging expectant mothers. It explores what the system should do from the point of view of midwives.

#### 2.2.3 Design and development

This next phase will be informed by the findings of the need assessment. It will involve the users to codesign a triage system, and it will draw its methodology from the design thinking steps engaging participants in a participatory workshop. Design thinking is part of the UCD approach. It prioritizes a more inclusive approach, placing deep empathy for end users at the center of design. It also prioritizes the desires, needs, and challenges of the end users to fully understand their problems in the hope of developing an effective solution (22, 23).

#### 2.2.4 Implementation and evaluation

The test and measure and post-test stage will be conducted upon finalization of the prototype. The authors envisage conducting a pilot testing of the intervention after its finalization.

### 2.3 Recruitment and data collection

In this study, midwives working in the district hospitals of Mpumalanga province were recruited to participate. Purposive sampling was used to select midwives working in the labor wards of maternity units. A piloted interview guide helped the authors to gain rich insights into the preferences of midwives in a midwife-led triage system. Data saturation was reached after 20 interviews, no new codes emerged after the 15th participant, and five more participants were interviewed to confirm data saturation. Thereafter, the data that emerged from the interviews were used to build a quantitative strand. A concise set of questions was developed to measure the preferences of midwives in the digital midwife-led triage system. The questions were rated using a Likert scale (Disagree, Uncertain, and Agree). The developed questionnaire underwent piloting and validation. It was reviewed and validated by experts in the field of midwifery and research. It was corrected and amended upon their requests. In the quantitative strand, the midwives were sampled using a stratified random sampling technique, and the participants were categorized into subgroups according to the hospitals to which they were attached. Questionnaires were distributed to the midwives and they were guided through the questionnaire completion process by the authors and field workers. In the quantitative strand, the sample size was calculated using a Slovin formula, it was found to be 171, but only 155 were completed and returned. The remaining questionnaires were attributed to incompleteness and non-participation by midwives. A detailed consent form was provided to all participants in both qualitative and quantitative strands.

*Sample size for midwives*

$$=\frac{N}{1+Ne^{2}}$$

$$n=\frac{300}{1+300{\left(0.05\right)}^{2}}$$

$$n=\frac{300}{1+300(0.0025)}$$

$$n=\frac{300}{1.75}$$

n=171

where, *n* = sample size; *N* = population size; *e* = error of margin (0.05).

### 2.4 Data analysis

The interviews were transcribed and analyzed following the steps of thematic analysis: (1) familiarization of the data by reading the transcripts multiple times, (2) generation of initial codes by identifying recurrent patterns and keywords, (3) search of themes—the authors grouped similar codes to identify themes and emerging subthemes, and (4) definition and naming of themes (24). The data were analyzed by the authors separately and then they reached a consensus on the emerging themes and subthemes. In the quantitative strand, the questionnaires were captured in Microsoft excel and analyzed using descriptive statistics with the assistance of the university biostatistician. Data were captured, cleaned, and analyzed using a Statistical Package for Social Sciences version 29.0. Frequencies and percentages were used to reflect the data. The reliability of the questionnaire was initially tested using Cronbach alpha. The Cronbach alpha of the questionnaire was 0.885, suggesting that the questionnaire is reliable and excellent, as proposed by Habidin et al. and Burns et al. (25, 26). The data were then integrated using a cross-case comparison analytical strategy to draw metainferences.

### 2.5 Methodological rigor

The authors followed the four principles of ensuring trustworthiness throughout the study, namely, credibility, dependability, transferability, and confirmability. This was achieved through various processes such as peer debriefing in conferences and prolonged engagement with midwives during data collection. Moreover, the authors adhered to the principles of purposive sampling. Meanwhile, the validity of the quantitative questionnaire was established through content and face validity. . Experts were involved in the process of content validity; the questionnaire was deemed readable and consistent. The Cronbach alpha of the questionnaire was 0.885, indicating that the questionnaire is reliable and excellent as indicated above.

## 3 Results

### 3.1 Demographic characteristics of the participants

The results highlighted that the majority of participants in both strands were women, aged between 40 and 49. Furthermore, the results highlighted that the majority were registered midwives holding a nursing diploma (R425) with a minimum of 1 year of experience in maternity units. This is shown in Table 1.

**Table 1:** Demographic characteristics of the participants, both strands.

Items	Qualitative	Quantitative
	Frequency, *N* (%)	Frequency, *N* (%)
Gender		
Male	1 (5)	20 (12.9)
Female	19 (95)	135 (87.1)
Age in years		
20–29	2 (10)	37 (23.9)
30–39	2 (10)	43 (27.7)
40–49	11 (55)	46 (29.7)
50–59	5 (25)	24 (15.5)
60–65	0	5 (3.2)
Occupation		
Advanced midwife	7 (35)	27 (17.4)
Midwife/Accoucheur	13 (65)	128 (82.6)
Highest level of education		
Bachelor's degree in Nursing (R425)	4 (20)	37 (24.0)
Diploma in Nursing (R425)	7 (35)	88 (57.1)
Advanced diploma in Midwifery	2 (10)	3 (1.9)
Post graduate/honors in Advanced midwifery	7 (35)	26 (16.9)
Years of experience in maternity unit in years		
1–5	6 (30)	68 (43.9)
6–10	3 (15)	42 (27.1)
11–15	5 (25)	25 (16.1)
≥15	6 (30)	20 (12.9)

**Table 2:** Themes and sub-themes.

Themes	Subthemes
1. Triage contents and features of the midwife-led triage system	1.1. Patient profiling and clinical history 1.2. Clinical profiling 1.3. Sufficient space to allow for current clinical assessment and fetal monitoring findings
2. Application and functionality features of the midwife-led triage system	2.1. Coupling of the devices and capability to share information and enhance communication among colleagues 2.2. Improved access to patient clinical data and results from ANC onwards by linking laboratory databases with digitized ANC records 2.3. Provision of guidance on expectant patient management after triage 2.4. Triage should have the potential to share pictures 2.5. A digitized questionnaire-style format should be used for the triage

### 3.2 Main results

This section presents qualitative and quantitative data. We conducted 20 individual interviews from May to July 2024 lasting for an average of 30–50 min. These interviews were supported by the quantitative surveys developed from the qualitative findings. Table 2 depicts the emerged themes and subthemes of the interviews.

#### 3.2.1 Qualitative results

##### 3.2.1.1 Theme 1: triage contents and features of the midwife-led triage system

The midwives identified several pieces of triage content that they wanted to be part of the digitalized systems. Midwives had diverse insights into an ideal digitalized triage system. The triage content included a patient profile, clinical profile, and designated space for a brief clinical assessment.

##### 3.2.1.2 Subtheme 1.1: Patient profiling and clinical history

Midwives expressed themselves on the ideal triage system that they preferred to have in their labor wards. Succinctly, midwives confidently stated that the triage system should include demographic and patient-related health information such as clinical histories. They stated that the information should include age, parity, gravidity, gestational age, and name. This was supported by the following assertions.

“I think what is important is the name of the patient, as well as surname, age, address, etc.” **P4**

“…obviously, the name…Name and surname. Age of the patient. And then gestational age.” **P8**

The midwives suggested the inclusion of the patient's history. This suggestion was based on their scientific knowledge of its impact on current conditions and patient management. Subsequent assertions supported the history suggestion. Taking a small pause, a midwife said;

“I think social history of the patient, the history of the environment in which she lives.” **P2**

Other midwives continued and added;

“Number 2 is the history that is subjective to what is happening.” **P13**

##### 3.2.1.3 Subtheme 1.2: Clinical profiling

In the realm of patient profiling, midwives suggested that this should be supported by clinical profiles. Gravidity, parity, gestational age, risk factors, vital signs, and clinical signs and symptoms of high-risk conditions should be included. The midwives noted the importance if of information such as gravity and gestational age. This was supported by the following narratives:

“So, you triage according to that. How severe is the pain, and the water as well, if perhaps she is ruptured, you would ask the colour of the water… Gestation as well, we should check it because you would find that someone else came in preterm labor so you have to triage her” **P1**

“We can include gestation, dilation, and also fetal heart rate. Actually, we need all of it, as well as parity because it is also important.. we can include age, parity, gravity and then gestation, as well as dilation and also the mode of delivery is important.” **P5**

Midwives prominently highlighted the determination of gestational age as a significant characteristic of the clinical profile. Gestational age falls under the parameters to be observed by midwives. Some midwives deem gestational age a vital parameter to determine, as it would inform them whether the patient is in preterm labor or not. Also, this guides the clinical management required by patients.

The inclusion of vital signs was supported by the following narratives:

“The vitals are what show us the BP of a patient first because other patients come here and say that they do not have hypertension only to find that when you check, it is very high. Therefore, vital signs are very important.” **P13**

“I suggested that all patients upon arrival should have vital signs, urinalysis, observations..  I don't know…” **P2**

“Maybe they can include vital signs that if a patient is like this, like if blood pressure exceeds this certain limit, she needs to be seen quickly.” **And** “also… I think they can include kind of signs of conditions that seem critical, maybe she's bleeding or experiencing epigastric pain or like that” **P17**

“Check vital signs, fetal heart it is after triaging. So, check any risk that you can point out, like, maybe, if the patient is bleeding or not, automatically that is triaging on itself. Also, check if the patient complains about severe contractions.” **P20**

Based on the midwives' feedback, they suggested that vaginal examinations, fetal heart rate, the color of the liquid upon rupture of membranes, and urinalysis are essential components of the clinical triage profile. However, it seems some of the midwives were not certain about their suggestions, as evidenced by the use of the phrase*, “I don't know.”* In addition, their suggested items form part of the clinical assessment of pregnant women who present on the labor ward. During triage, the midwives considered it mandatory to assess these factors.

##### 3.2.1.4 Subtheme 1.3: Sufficient space to allow for current clinical assessment and fetal monitoring findings

The midwives mentioned that another important aspect that is necessary for a digitalized triage system is that the triage system should have adequate space for documentation. This will help them to write and enter patient's clinical findings and main complaints.

“And then I must enter, there is a space where I enter my findings.. I will just write what the patient is currently presenting with. The current one from the history that I will be collecting, like what is bringing you here, what are you feeling? Okay. Yeah, that's the only thing I will enter.” **P3**

“The app should give me a space to fill in.. a small space for description where I will get to present my patient on what happened and also the outcome.” **P5**

The enthusiasm of midwives in designing the triage system shows their willingness to use it. As mentioned in the extracts, one of the midwives strongly emphasized that she must be able to enter her findings within the system.

##### 3.2.1.5 Theme 2: application and functionality features of the midwife-led triage system

The midwives confidently voiced their specific requirements for a digitalized triage system. Some of the functionality triage features were related to their real-life experiences that they are currently struggling with, and they sought to outline features such as accessibility of information, the linking of devices to enhance communication between colleagues, and the potential of a digitalized triage system to take pictures.

##### 3.2.1.6 Subtheme 2.1: Coupling of devices and capability to share information and enhance communications among colleagues

The linking of devices and communication was identified by the midwives as a huge functionality aspect needed in the digitalized triage system. The functionality to share information with other healthcare personnel was the prominent subtheme of the study, as they stated that sharing information improves communication. The midwives indicated that the triage system should allow the use of cell phone devices to enhance the communication between them and the doctors when there is an emergency. This was supported by the following quotes:

“…mmh, I don't know how to put it.. it should be a system where we will be able to communicate with others, it should definitely not be a robot…” **P10**

“I think you should share. Maybe with the next person only. Maybe.. with a person in the hierarchy, with the doctor, or share with the senior nurse. Or share with what? I don't know. Isn't you looking for a way forward for that patient? You share with someone who is there to help.” **P8**

Another midwife experienced with digitalized systems elaborated further to say,

“, yeah, it must include that sharing. I can share it, it is very true. Like where I used to work, you know, you would send to, you call it Euclid, it's European Clinical Data System. So, when you finished with everything you took from the machine that you were using and what I was busy with or my findings that I was typing in, I would send them to the operational manager on the ward and to the head office and to the doctor, our clinical doctor, there was a feature where you classify if it is emergency or just normal, you know. So they will know that we have an emergency for such. It is just that I am not a technical person. I don't know how they did that, but you were able to pick up, like after picking, after reading, or putting the patient in the machine, you type your history and then you classify the patient. If it is an emergency or for the doctor's attention or normal…” **P3**

The enthusiasm for this functionality feature was related to the failure of reaching other healthcare personnel during emergencies due to switchboard malfunctions. Midwives were concerned that they were sometimes unable to reach doctors after triaging the patients. Thus, in the presence of a malfunctioning switchboard, they strongly expressed the need for the digitalized system to have the ability to be connected to other devices to enhance communication.

##### 3.2.1.7 Subtheme 2.2: Enhanced access to patient clinical data and results through integrated laboratory databases and digitized ANC records

Several midwives relayed the clinical complexities involved when triaging patients. One of the complexities was the struggle to access patients' results upon triaging in the labor ward. Therefore, the midwives strongly suggested that the digitalized triage system should be linked to laboratories and digitalized ANC records to enhance accessibility to patient clinical data and results. This was supported by the following assertions:

“I am still thinking that it should have access to the results because we are struggling to access the results when a patient arrives” **P14**

“What can I say, for example, if a patient was attended at casualty during pregnancy and now comes to the labor ward, we should be able to access that information in the system and it would be easier for us to access that information from the clinic, casualty. We will be…there will be no information gaps…” **P5**

According to the midwives, the primary reason behind the need for improved access to clinical data and patient results from antenatal care is that, in certain cases, local clinics fail to document patient booking results and they struggle to access to the results. Patients also tend to lose their antenatal maternity records and, when this happens, they have to start afresh. Therefore, digitalized antenatal care was considered a better option to enhance the accessibility of antenatal care data and results. Concerned about the practices of midwives in local clinics, a midwife said,

“…because we come across patients who in the local clinic don't review the results. Especially if they attend clinics more than 10 times. They don't have Resus factor (RH) and syphilis (RPR) results. It's like they don't know the importance of those things.” **P8**

##### 3.2.1.8 Subtheme 2.3: provision of guidance on expectant patient management upon triage

Midwives prioritized the need for a digitized triage system that provides clinical management guidance aligned with established maternal health guidelines. Maternal guidelines should be incorporated into the triage system. The midwives said

“…with the guidelines, I think they need more guidelines on our side as midwives that will help us to treat patients immediately and also to avoid errors and malpractices in some cases. So, if we have like, we could have like certain guidelines or add to the ones that we put before, but in a more advanced way or time efficient manner, that would help us a lot…yeah, the maternal guidelines should be incorporated to the system, yeah. It should be incorporated into the system, also into the software, yeah, that it would like to assist you at that moment when you are dealing with that patient, that this should be done in such a way. It should be incorporated in every situation” **P11**

“It should also have guidelines, for example, if a patient with eclampsia arrives, it should state how we treat eclampsia. HELLP syndrome guideline because sometimes you only find that the condition needs to be managed by an advanced midwife and at that particular time the advanced midwife is not there, and I do not know how to manage this condition” **P14**

This was a common concern among midwives. With concern in her voice and dispirited, a midwife further said. “..when we discuss with the patient files and the PPIP (Perinatal mortality meetings), you can see that most of the problems were just overlooked. You ask yourself: What happened? Is this person really aware? Because you see the person wrote it down, it is there but failed to act” **P8**

##### 3.2.1.9 Subtheme 2.4: the triage should have the potential to share pictures

The midwives felt that the ability of the digitalized triage system to take pictures would be a beneficial feature. The need for the picture feature on the digitalized system was exemplified by the subsequent quotes:

“It should have the feature for pictures…” **P6**

Followed by the probing question “Why?”

“We should be able to see what we are dealing with and be able to share it also” **P6**

Another participant said,

“It should include a picture of the patient or a picture of the patient's condition” **P2**

**Probing question; “when you say patient picture, do you mean clinical picture?”**

“Like a real picture. For example, if a patient has an ear infection, you take it and share it with other colleagues. I don't know if that's allowed.” **P2**

“I think we can have a picture of the pregnant woman and then patients who have had complications we can also include them because we don't know what will happen to that patient” **P4**

Drawing from the extracts, it seems that the midwives want the digitalized system to have the capability to take pictures. However, their justification for this is not properly explained. It can only be assumed that it is needed for evidence-keeping and consultation with other colleagues, as one midwife mentioned the digitalized triage system should be able to share pictures with colleagues.

##### 3.2.1.10 Subtheme 2.5: a digitized questionnaire-style format should be used for the triage

Another key functionality feature that was prominently indicated by the midwives was that the digitized triage system should adopt a questionnaire-style format. This was supported by the subsequent quotes:

“…as well as a place where we would get questions with answers like yes or no. So, at least, it would be easier than capturing and asking questions. Mostly, it should have questions that are closed-ended, like, for example, in gender we have male or female so it should also include that and I would just tick unlike writing that from scratch.” **P18**

“Like as I've already explained, we need some sort of questionnaire that you put there so that when we ask the patient when and how, how many children does she have, age, etc. What comes first is the examination of the patient from head to toe, like those things.” **P12**

The midwives hoped that the digitized triage system would reduce how much writing they had to do and it should not add to their currently existing workload as it will make things difficult for them.

#### 3.2.2 Quantitative results

The findings in Table 3 indicate that the majority of midwives agreed that the digitalized systems should include the patient's name (96.1%), age (96.1%), risk factors (94.8%), clinical history (96.8%), the current condition of the patient and the main complaint (94.8%), parity (95.5%), and gravidity (95.5%). Of the midwives, 85.8% indicated that the digitalized triage system should have an area designated to write their subjective data and the patient's existing problems and 90.3% of midwives indicated that the digitalized system should incorporate maternal guidelines. Another large percentage of midwives agreed that the digitalized system should provide guidance on patient management (94.2%), be connected to doctors on call so they can get in touch with them immediately (91.6%), be connected to laboratories to improve access patient results (95.5%), and be implemented in local clinics to enable access to patient information from antenatal care booking (96.1%). Furthermore, most of the midwives agreed that the digitalized triage system should have functionality to capture and transmit pictures (75.5%) and be in the form of a digital questionnaire (87.7%). This highlights that midwives are cognizant of their needs and preferences for the digitalized triage system. The Cronbach alpha of the constructs in the codesigning section was 0.885, suggesting that the constructs were good and reliable.

**Table 3:** Quantitative results.

Item		Disagree	Uncertain	Agree	Mean	SD
		*N* (%)	*N* (%)	*N* (%)		
D1	I suggest that the patient's name should be included in the triage system	3 (1.9)	3 (1.9)	148 (96.1)	2.94	0.308
D2	I suggest that the patient's age should be included in the triage system	2 (1.3)	4 (2.6)	149 (96.1)	2.95	0.275
D3	I suggest that the patient's risk factors should be included in the triage system	4 (2.6)	4 (2.6)	147 (94.8)	2.92	0.353
D4	I suggest that the patient's current condition and chief complaint should be part of the triage system	3 (1.9)	5 (3.2)	147 (94.8)	2.93	0.326
D5	I suggest that the patient's parity should form part of the triage system	3 (1.9)	4 (2.6)	148 (95.5)	2.94	0.317
D6	I suggest that the triage system should also include the patient's gravidity	3 (1.9)	4 (2.6)	148 (95.5)	2.94	0.317
D7	Clinical history (Medical, gynecological, and surgical)	3 (1.9)	2 (1.3)	150 (96.8)	2.95	0.298
D8	Area designated for examination and the patient's existing issues	9 (5.8)	13 (8.4)	133 (85.8)	2.81	0.508
D9	Incorporated and consistent with maternal guidelines	3 (1.9)	12 (7.7)	140 (90.3)	2.88	0.378
D10	Give advice on patient management.	2 (1.3)	7 (4.5)	146 (94.2)	2.93	0.305
D11	Connected to doctors on call so we can get in touch with them right away	6 (3.9)	7 (4.5)	142 (91.6)	2.88	0.433
D12	Linked to laboratories, so we can access patients results	1 (0.6)	6 (3.9)	148 (95.5)	2.95	0.250
D13	Linked to fellow colleagues, so we can share patient information	14 (9.0)	6 (3.9)	135 (87.1)	2.78	0.597
D14	Implemented at local clinics, so we can access patient information from antenatal care booking	3 (1.9)	3 (1.9)	149 (96.1)	2.94	0.308
D15	The functionality to capture and transmit pictures	14 (9.0)	24 (15.5)	117 (75.5)	2.66	0.639
D16	It needs to be in the form of a digital questionnaire.	8 (5.2)	11 (7.1)	136 (87.7)	2.82	0.500
Cronbach alpha		0.885				

#### 3.2.3 Mixed methods results

A mixed-method cross-case comparison analytical strategy underpinned the analysis of the study. A joint display was used for presentation to characterize the integration and comparison of the qualitative and quantitative data to gather new insights beyond the data by the qualitative and quantitative data as single research methods. The integration of data from the qualitative and quantitative strands generated a total of two confirmed findings. The confirmed findings included triage contents and features of the midwife-led triage system as well as application and functionality features. Table 4 below shows the joint display of the integration process of the qualitative and quantitative strands.

**Table 4:** Joint display of the integration process of the qualitative and quantitative strands.

Principal themes	Qualitative results	Quantitative results
Triage contents and features of the midwife-led triage system	The midwives shared several suggestions on what the digitalized system should entail. The Midwives mentioned triage contents such as the patient's profile, the clinical profile, and the clinical history “*…obviously, the name…Name and surname. Age of the patient. And then gestational age.” **P8*** “*…also, family history…is there any family history in a family because it can be passed down from generation to generation….” **P19*** “*Number 2 is the history, that is subjective to what is happening” **P13***	In total, 94–99% of the midwives responded agree that digitalization of the triage system should include the clinical profile, patient profile, and clinical history
Mixed method meta-inferences	Confirmed. Qualitative responses were consistent with quantitative surveys that factors such as patient name, age, risk factors, parity, and gravidity should form part of the triage system. The factors identified by the midwives were related to the potential of each demographic factor. For instance, the midwives reported that noting the patient gravidity and age allows them to identify whether the patient is high or low risk	
Application and functionality features of the midwife-led triage system	The midwives shared several suggestions on functionality and application features of the digitalized triage system. Supporting quotes; “*, yeah, it must include that sharing. I can share…” **P3*** “*I am still thinking, it should have access to results because we struggle to access results when a patient arrives” **P14*** “*Yes. It should guide you; it should guide anyone seeing the patient and be alert…” **P8***	In total 75–96% of the midwives indicated that the digitalized triage system should have triage functionality and application features such as information sharing and enhanced access to laboratory results that are incorporated and consistent with the digitalized triage system
**Mixed method meta-inferences**	**Confirmed.** The midwives were consistent with the quantitative data that the triage functionality and application features such as information sharing, emergency contact of doctors on call, and incorporation with maternal guidelines should be included in the triage system. Some of the midwives identified features such as guidance on patient management, sharing of pictures, and questionnaire format as some of the key features of the digitalized triage system	

P(n), participant number.

## 4 Discussion

**Figure 2:**
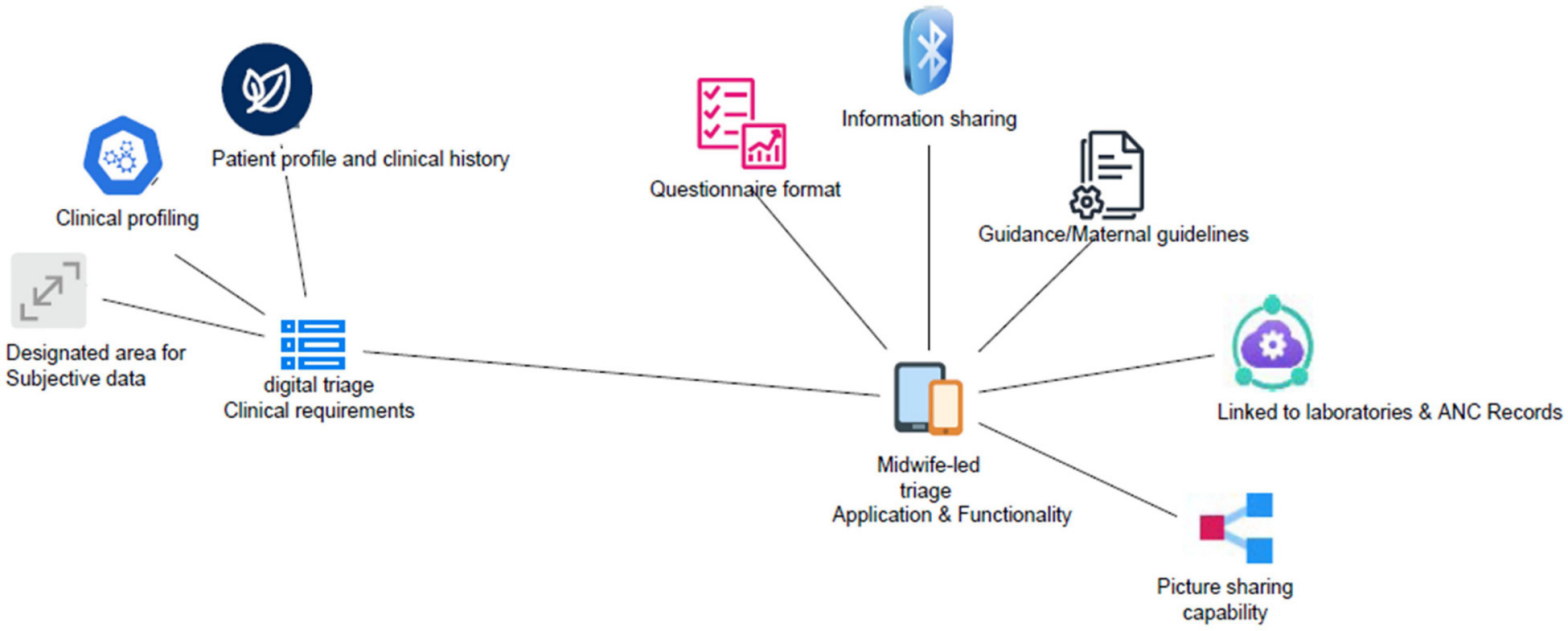
Empirical findings.

This contemporary study ventures into codesigning a triage system with midwives for midwives. Its interests lie solely in identifying the needs and preferences of midwives of a digital triage system as a first step to codesign. The study identified that midwives have vivid preferences for digital triage systems. Their preferences were shaped by their current experiences within the workplace and clinical environment. The findings are presented in Figure 2. The main themes and key trends are discussed below.

### 4.1 Triage contents and features of the midwife-led triage system

The integrated findings succinctly highlighted the key triage contents and features that should be included in a digitalized triage system. The identified user needs and preferences were patient profile, clinical profile, and designated space for clinical information. Drawing from the preferences of the end users, they indicated that things such as age, parity, and gravidity are crucial to diagnosing and noting whether a pregnant woman is high risk or not. For instance, one of the end users in the qualitative strand indicated that gravidity and age play a crucial role in identifying whether the pregnant women needs urgent attention or not. For example, mothers with more than five children are considered high risk. The midwives related the importance of patient profile to the determination of the specific care the patient needs. It was strongly believed that items such as age in triaging matter the most because this allows midwives to determine whether a particular patient is high-risk or not. For example, a midwife further articulated that age is important because you will be able to know if that patient is an advanced maternal age or not. According to midwives, variables such as age and gravidity have an impact on how care is provided to pregnant women, and clinical management is not the same. Nekkanti et al., reports pregnant women can be vulnerable to unforeseen complications that could harm the woman or the baby. Complications could be precipitated by factors such as childbearing age, nutritional status, education level, and access to healthcare (27). A study conducted in China agreed that pregnant women with advanced bearing age, primiparity, obesity, and low educational level are at increased risk for pregnancy-related complications (28). Therefore, midwives were concerned with the variables of the patient's clinical profile.

The empirical study also discovered that midwives need support such as resources and training. It seems that midwives were cognizant to what is required to ensure widespread implementation of the digital triage system. They expressed concern regarding how they would comply with a digital triage system if it were developed and implemented without providing them with the necessary training. This indicates the intention to use them if they are trained. According to Parajuli et al., training plays a pivotal role in improving awareness and widespread implementation of digital health interventions (29). Therefore, midwives should receive training to scale up their triage skills to improve the sustainability and scalability of digital health interventions.

### 4.2 Application and functionality features of the midwife-led triage system

In this aspect, the qualitative responses confirmed the survey responses. Midwives indicated that the digitalized triage system should have functionality features such as sharing and should be incorporated with maternal guidelines and provide guidance on patient management. Drawing from the experience of midwives, concerns about the provision of guidance in the management of patients by midwives were related to the shortage of midwives with advanced skills and competence in the management of obstetric emergencies. In this study, some midwives were concerned with the skills of other midwives in the management of obstetric conditions. The findings also highlighted that some midwives lack a sense of urgency and the ability to provide efficient and quality obstetric care. Existing evidence demonstrates that the use of maternal guidelines improves and promotes the provision of quality maternal care. Maternal guidelines provide guidance on how obstetric conditions should be treated (30). The midwives in this study shared similar concerns with the study of Sethi et al.*,* which indicated that midwives lack knowledge of the evidence-based maternal guidelines for managing pregnant women presenting to maternity units (31). In support of these findings, Ramavhoya et al.*,* highlighted that midwives have a knowledge deficit of the maternal guidelines (30). In existing South African maternity guidelines, obstetric triage is superficially described and does not detail how triage should be done and implemented by midwives. From the feedback of midwives in this study and the existing literature, it is evident that this calls for urgent action for daily in-ward training of midwives to equip them with skills to handle obstetric emergencies and an actionable national strategy aimed at improving the knowledge and competency of midwives. Furthermore, a digital triage system should be developed incorporating maternal guidelines to provide guidance to midwives in the management of obstetric emergencies.

Midwives also highlighted that the digitalized triage system should be able to take and send pictures and be in questionnaire format. Based on the feedback from midwives, these functionality features will reduce workload and enable them to share information with their fellow colleagues. Bromwich et al. indicated that the use of mobile phones for healthcare communication is an effective tool for professional interaction and facilitates easier access to decision support. However, it is associated with a privacy risk (32). Shigekawa et al.*,* conceded that telecommunications are associated with legal and privacy issues, although it has the potential to improve some patient outcomes and reduce costs of healthcare. This indicates that digital health interventions must comply with ethical and legal aspects of health care (33). This could be achieved by using encryption. Zegeye suggests that a three-way authentication process should be adopted using pin, password, and biometric fingerprints to access patient information (34). With midwives' concerns about privacy and security, data protection and handling sensitive patient information remains a mandatory practice. For example, a study by Conduah et al. highlights that breach of privacy and security in digital health interventions is a challenge across North America, Asia-pacific, Europe, and Sub-Saharan Africa. The scholars emphasize the leveraging of artificial intelligence and machine learning algorithms to detect breaches of privacy and enhance data protection and encryption of patient information (35). Furthermore, similar to Zegeye, this study suggests that legal and regulatory frameworks and policies regarding digital health interventions should be developed to govern privacy and security matters when using digital health interventions (34).

Overall, based on the findings of the study, midwives indicated that they face resource challenges, and this hindered them in providing quality triage. Therefore, they strongly believe that this triage innovation will help improve quality care and proper allocation of resources. This indicates that the availability of resources and tools shapes the needs of midwives in patient care. In simple terms, things like resources and tools play a crucial role in our collective lives. This suggests that understanding the needs and preferences of midwives is crucial to facilitate the development and implementation of innovations such as digital triage in the maternity units in South Africa. This calls researchers and policy makers to take into consideration the needs and preferences of healthcare providers, as this shapes how care is provided to patients.

This study has contextual limitations, as the study was carried out only in district hospitals in Mpumalanga province. Therefore, the results cannot be generalized to other contexts. This suggests that studies should be conducted in other regions to gather comprehensive understanding of the phenomenon under investigation. Although the study followed a UCD approach, only the requirement-gathering phase is reported in this study. This study only reports on the preferences of the midwife-led triage system and did not explore any feasibility considerations of the midwife-led triage system. Therefore, future research should consider the development of a digital triage system and integrate it with users' suggested features. In addition, due to the exploratory nature of the study, the study focused on exploring the needs and preferences of the midwives rather than measuring the causality and effect associations of the variable. Future research should attempt to explain causality and effect associations between the demographic data and triage features indicated by the midwives. Most importantly, this study relied on self-reported data, subjecting the study to social desirability bias.

However, the study draws its strengths from the adoption of the UCD and Mixed Method approaches in this paper. The UCD methodology in this paper focused mainly on deep interaction with the target users to create digital and technological solutions that are grounded in the information about the people who will be using them and the context in which they might be deployed (16, 36). Embedding the sequential exploratory research design within the UCD enhanced the comprehensiveness of the data collection.

## 5 Conclusions

In conclusion, this paper discussed the preferences of midwives for a digital triage system. It appears that midwives' preferences are shaped by their experiences and the existing environment. Therefore, this paper suggests that the preferences, needs, and ideas of midwives should be included in a new quality triage system for South Africa's maternity units. Furthermore, the relevant stakeholder should provide the necessary resources to navigate a smooth triage process for patients with the midwife-led triage system.

## Data Availability

The raw data supporting the conclusions of this article will be made available by the authors without undue reservation.
